# The impact of shelter-in-place during the COVID-19 pandemic on social support for mental health recovery: A prescribing-oriented qualitative study of patient perspectives

**DOI:** 10.1371/journal.pone.0316582

**Published:** 2025-01-17

**Authors:** A. Song, T. Chao, B. Harris, H. Hsin

**Affiliations:** Kaiser Permanente San Jose Psychiatry, San Jose, California, United States of America; University of Helsinki, FINLAND

## Abstract

The COVID-19 pandemic created unprecedented challenges for social connectivity and mental health, especially during mandated shelter-in-place periods. For patients engaged in mental health treatment, the social impact of their shelter-in-place experience remains an area of active investigation. This is particularly relevant in the context of social prescribing, a growing area of clinical intervention where healthcare providers actively refer patients to local social resources or activities to enhance mental health and wellbeing. Here, we investigated patient perspectives on their social supports during shelter-in-place, with an eye toward informing future social prescribing and counseling steps that mental health clinicians can take to foster social resiliency of patients. We conducted semi-structured phone interviews with 12 participants receiving mental health treatment at Kaiser Permanente San Jose Adult Psychiatry clinics. Interviews were transcribed and analyzed for themes. The sample consisted of 8 female and 4 male participants between the ages of 21 to 44, who received services including outpatient medication management, outpatient therapy, and/or outpatient group therapy. Analysis revealed two thematic categories that participants identified: (A) Specific ways that shelter-in-place impacted their social experience and (B) specific types of social relationships that participants felt were important to their mental health and wellbeing. In the first category, thematic factors that affected the social experience included (1) COVID-19-related health concerns, (2) participants’ baseline socialization patterns (degree of introversion/extroversion) and (3) the use of online and social media communication channels. For the second category of themes, specific functions of social relationships identified included those centered around (a) instrumental support, (b) emotional support, (c) community connection. Many relationships served more than one function during shelter-in-place, and many participants cited perceived community connection as a particularly important element in their mental health recovery. These themes highlight key contributors to the social experience of the shelter-in-place period for individuals in mental health recovery and inform future ways that clinicians can structure social prescribing practices to better assist the needs of patients.

## Introduction

The COVID-19 pandemic and associated policies in social distancing were unprecedented in scale. Contact restrictions imposed due to concerns of COVID-19 spread introduced risks of isolation and loneliness, which can be particularly difficult for individuals struggling with mental health symptoms, or at risk of mental health decompensation. Recent survey studies have shown associations between social distancing behavior and worsened anxiety and depression symptoms [1]. In a survey conducted among 666 youths aged 16–25, psychological distress was progressively more likely to occur as levels of social isolation increased during the COVID-19 pandemic [2]. Higher perceived social support during the pandemic, by contrast, was associated with lower risk of depression and improved quality of sleep [3] and better coping skills [4]. A study investigating the impact of the COVID-19 pandemic on 247 adolescents’ mental health found that the feeling of social connection during the lockdown protected against poor mental health [5]. Lastly, longitudinal data from over 69,000 participants of the US National Institutes of Health *All of Us* research program showed that social support during the COVID-19 pandemic was associated with lowered odds of depression symptoms [6]. Similar results have been shown in studies taking place in other countries [7, 8].

Engagement in social and leisure activities have been shown to improve mental health symptoms of anxiety, depression, bipolar disorder, and schizophrenia [9]. Many studies have demonstrated positive correlation between perceived social support and decreased risk of anxiety and depression [10, 11]. Loneliness and social isolation have also become increasingly recognized as public health risks and drivers of adverse health outcomes [12]. As a potential response to these concerns, social prescribing (SP) has emerged as a modern intervention within healthcare systems [13]. Commonly used in the primary care setting, SP allows the provider to incorporate non-clinical resources such as community resources, support groups, exercise and music classes, as well as other aspects of the patients’ existing support network, to engage patients in their recovery and wellness [13]. The Plus Social program in Australia and the INSPYRE program in the United Kingdom are examples of SP programs that have improved biopsychosocial outcomes in individuals with mood and psychotic spectrum disorders [13]. Despite its potential, SP remains under-utilized in the United States, especially in mental healthcare settings [12].

To date, there has been little qualitative investigation into how individuals with mental health challenges experienced the isolating social landscape during the COVID-19 pandemic. In this study, we sought to understand the ways in which individuals navigated the early shelter-in-place period of the pandemic, with an emphasis on their experience of social connection. Our goal was to identify gaps in social needs that arose during the shelter-in-place period, with the intention of informing potential approaches to boosting social resiliency among mental health patients in future pandemic crises. As SP practices begin to trickle into mental health care settings, we also saw an opportunity to shape the clinical context of SP by identifying social needs and experiences that patients identified as crucial to their wellbeing during a period of intense social isolation.

## Methods

This qualitative research study was conducted using a phenomenology-driven approach in order to better understand the meaning participants ascribed to their social experiences during the pandemic [14]. The study was conducted within the Adult Psychiatry department at Kaiser Permanente (KP) San Jose in Northern California, a large, integrated healthcare center that serves a diverse population representative of the surrounding communities. During the COVID-19 pandemic, the state of California instituted mandatory shelter-in-place policies from March 2020 to January 2021 that limited the social contacts and required masking for in-person activities [15]. During this period, the clinical department adopted rapid measures to continue serving patients, including the widespread adoption of telehealth services. Clinic leaders supported a qualitative study aimed at exploring how patients’ social supports changed over the pandemic’s shelter-in-place period. The Kaiser Permanente Northern California Institutional Review Board (IRB) determined this study to be exempt.

### a. Participants

We identified 30,133 patients from electronic health records who attended a mental health appointment (virtual or in-person) at Kaiser Permanente San Jose Adult Psychiatry within the 12 months preceding the study. Of this group, 650 potentially eligible participants were randomly selected by a random number generator. To be considered eligible, participants needed to be between the ages of 18 to 44 years, an inclusion criterion selected to focus on a generation of patients who were comfortable with social media, which we wished to inquire given its increasing adoption as a modality of social connection. Additional inclusion criteria included participants receiving concurrent care with a KP San Jose mental health clinician at the time of the study, having the ability to read, speak, and write conversational English, being able to receive messages requesting participation from the Kaiser Permanente online patient portal, and being physically present in the Northern California Bay Area during the eligibility screen and the study interview. These criteria were chosen to align with our recruitment and interview process, which began with an invitation letter written in English and sent through the online portal, and ended with a phone interview conducted in English, the primary language of the study team members. Exclusion criteria comprised of employment by Kaiser Permanente or its affiliates, active suicidal or homicidal ideation (but having prior ideation would be acceptable), concurrent treatment in an intensive outpatient program or other higher level of care (but having prior treatment at higher levels of care was acceptable), active conservatorship or guardianship by another individual or state wardship, receiving direct clinical services during study participation from any member of the clinical study team, or by investigator discretion.

**Fig 1:**
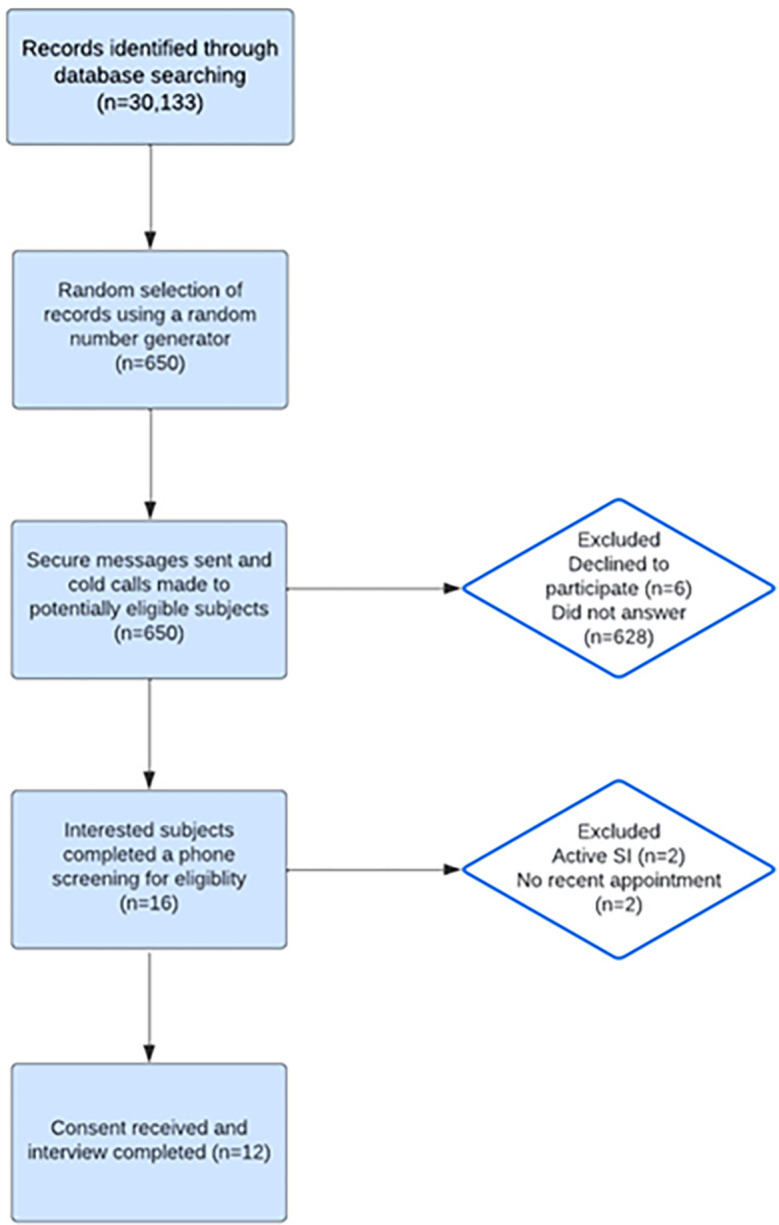
CONSORT diagram. Eligibility screening and enrollment.

The 650 potentially eligible participants were sent a secure message through the online patient portal inviting voluntary participation in the study. Approximately 100 of these potentially eligible participants also received a phone call from a study team member to invite participation. Of the 650, six individuals declined participation and 628 individuals did not respond to either secure message or phone call. Those who declined to participate cited reasons such as lack of time or interest in being involved. Sixteen subjects expressed interest and completed the eligibility phone screening interview. Two of the subjects screened were excluded due to having active suicidal ideation, and two were excluded due to not having a current mental health provider in the department. Twelve remaining eligible patients were verbally consented (as documented and witnessed by the study team; wavier of written consent was submitted to the Institutional Review Board) and completed the study interview (Fig 1). Recruitment occurred from 1/6/2023 to 3/3/2023.

### b. Procedure

To enroll patients, the study team contacted a random selection of 650 potentially eligible patients by phone or secure message. Secure messages included a succinct description of the study, ways to contact the study team if interested in participating in the study, and the option to opt-out of further contact from the study team. An information sheet (S2 Fig) was attached to the secure messages and provided more details about the study, such as informed consent and privacy. Subjects who expressed interest completed a phone eligibility screen. Subsequently, the eligible subjects were scheduled for a phone appointment to complete the verbal consent process, to ask any further questions or opt-out of the study if they wished, and if enrolled, to participate in completing the study. Interviews were semi-structured (see S3 Fig for interview guide), lasted approximately 45 to 60 minutes, and were conducted by two clinical psychiatrists (TC and AS). Upon the interview’s completion, participants were eligible for compensation of Kaiser Permanente branded gift items worth about $30. Enrollment continued on a rolling basis concurrently with data analysis, until the research team deemed that saturation of themes had been achieved. Twelve participants completed the study. The interviews were recorded on Microsoft Teams software, with all communications and data storage occurring behind the Kaiser Permanente firewall. Research data was stored without any personally identifying information of participants. Interview recordings were transcribed by a study psychiatrist with the aid of Microsoft Teams transcription software and reviewed for quality control purposes by a second study psychiatrist before analysis.

### c. Data analysis

Study clinicians used sensitizing concepts to guide examination of transcript data through an open, inductive approach, as described previously [16]. Each participant’s transcript was coded independently by two study psychiatrists (TC and AS), who then met and consolidated codes for each participant by consensus. Any specific social supports, social experiences, and/or social connection modalities mentioned were highlighted as potential codes in initial review of transcripts, as well as any experiences of the COVID-19 pandemic on social experiences or mental health. Ideas for how to “prepare socially” for a future pandemic were also coded. All codes were reviewed to find associations or recurring patterns among subjects’ answers that could serve as independent themes. Themes were identified from the coding library and consolidated by both study psychiatrists in discussion with a third study psychiatrist (HH). Quotes were maintained throughout the entirety of analysis to encapsulate the essence of the topic being discussed, relevant emotions, or experiences at hand. When codes from new interviews began consistently mapping to previously identified themes, saturation was considered achieved and study enrollment concluded. Reflexivity was maintained through reflection meetings between study team members throughout interviewing and analysis steps. Our analysis followed SRQR (Standards for Reporting Qualitative Research) guidelines [17].

## Results

The sample comprised of 8 self-identified female and 4 self-identified male participants between the ages of 21 to 44. Self-reported race/ethnicity of participants included African American, Hispanic/Latinx and White. Self-reported education levels included high school, some college, college, and postgraduate degrees. Services that participants reported receiving included outpatient medication management, outpatient therapy, and outpatient group therapy. We found two dominant thematic categories during analysis: Category (A) included factors that influenced the social experience during shelter-in-place, and Category (B) included types of social relationships that were identified as important to participants’ mental health and wellbeing. Within the first category, we found three themes: (1) individuals’ baseline socialization patterns, (2) health concerns related to COVID-19, and (3) use of electronic and social media communication channels all heavily impacted the social experience. Within the second category, we found three types of social supports that participants felt were helpful to their mental health: (1) instrumental support, as in social connections that provided material or logistic needs, (2) emotional support, as in personal interactions that helped participants feel connected to someone, and (3) community connections, as in collective bonds that gave participants a sense of belonging.

### Thematic category A: Factors that influenced the social experience of shelter-in-place

#### 1. Baseline socialization patterns of individuals

Participants who considered themselves more introverted at baseline reported feeling more comfortable with the decreased social exposure associated with the pandemic, compared to those who considered themselves more extroverted. Many participants talked about how comfortable they felt when they did not feel the pressure to socialize as much.

“I *think I was actually pretty happy during the pandemic… I tend to have agoraphobia… I just stayed in the house and I didn’t leave, which to me was so relaxing… because sometimes just getting up and going out and seeing people is terrifying. Going into crowded places is sometimes hard… I knew I didn’t have the pressure of having to go somewhere.”*

Some participants considered themselves more extroverted at baseline, and expressed sadness that the pandemic had made accessing their social connections more challenging.

“*It wasn’t that much of a change in some aspects, because I’d already gotten used to having most of my good friends be at least three-and-a-half hours away from me, so I had started to get accustomed to a more text- or call- based friendship, but it was really rough for me. I like being around people. And it was kind of hard to not have that option anymore. I didn’t really realize how much I relied on the in-person socialization until it happened.”*

Beyond personality traits, some participants cited their stage of life as a factor in how distressed they felt as a result of social isolation. The “baseline” of anticipated phase-of-life changes were affected for these participants, who felt as if their lives were put on hold because of the pandemic, creating a sense of lost time. This theme did not come up as much for individuals who had already built a strong network of interpersonal relationships prior to the pandemic.

*“I’ve always been kind of a late bloomer so I felt like the last little remnants of being able to hang out as a fun, single adult were taken from me and it took me a while to find this current boyfriend and I had two years. Maybe I could have found them if this pandemic was, you know, not here. And I could already maybe married. And I want kids, and so maybe that could have happened two years earlier than it did. So I do have a little bit of resentment towards all the shelter in place things.”*

#### 2. Health concerns related to COVID-19

Many individuals described their concern of contracting COVID-19 and the resulting potential health costs as a key contributor to their social experience during the pandemic. This was particularly felt in relation to concern for the health of family members, which often served as a powerful motive to minimize social interactions.

“*And especially my youngest is [immuno]compromised so I didn’t want to risk anything unnecessarily.”*

“*And I was trying to be very careful*. *My mom has an autoimmune disease*, *so we were like ‘no contact’ with most people*.*”*

#### 3. Use of online and social media communication

Participants mentioned the difficulty of adjusting to virtual forms of communication when they were accustomed to meeting in-person. Some found it difficult to make the switch while balancing the needs of their families day-to-day.

“*Having to kind of connect with my friends during the pandemic was actually pretty hard because we like to get together once a while and go for breakfast or go for lunch and you know, we weren’t able to do that. So calling each other over the phone was kind of hard … because we had our own families that we were trying to take care of during the pandemic in the beginning.”*

Participants reported a wide spectrum of engagement with social media and thoughts on how useful social media was for them during shelter-in-place. Some knew that it was detrimental to their wellbeing and stayed away from it.

*“I’ve since purged certain people that I follow. I’d like to think my social media is pretty basic at this point where I’m not… following anybody who puts these things out there that can change my mentality towards myself."*

*“[Social media was] not always I found ‘for’ my mental health*. *It’s not good to be on those things*. *So I haven’t been*.*”*

Others embraced social media as a way of finding community and even motivating themselves to pursue hobbies.

“*I relied on Instagram. I started to notice I followed more creative people and so it led me to find an art group and so I began to follow on Instagram an artist from [another state] that painted one hundred days of art tutorial… so I started to paint more and create more… and that was one of the things that helped me. “*

Together, these results identify several primary factors (baseline socialization patterns, health concerns of COVID-19 spread, and social media uses) that appeared to influence participants’ social experience during shelter-in-place.

### Thematic category B: Types of social support

As participants cited examples of social connections that were helpful to their mental health, three themes of social support were identified:

#### 1. Instrumental support

Many participants noted that support with finances and financial stability, as well as support with material necessities such as groceries, were helpful during the shelter-in-place period.

“*I remember my sister helping me… she would go to the store to get groceries and she would bring some over. And then our neighbors… worked in the hospital setting so I remember he gave us some supplies that he had, so he definitely helped us with more of the [personal protection equipment].”*

“I *was working a lot and I have my two girls*. *It was just a single income household and*… *I had good work friends*… *friends that became really close to me*. *They actually helped me a lot with my children during that time right before the pandemic*… *whether they were with my children or supervising*.*”*

#### 2. Emotional support

We found that all participants who described an overall positive narrative of their social experience during the pandemic expressed significant feelings of closeness and intimacy with a small group of individuals.

“*Now [during the pandemic] we’re living together twenty-four/seven. I thought it was the greatest thing ever. I thought being home with my family, just me and the kids and my wife and my parents was a dream come true.”*

“*If it’s one person*, *three*, *four*, *whatever it is your social supportive network*. *It doesn’t have to be a lot of people*. *Just a few people to count on makes a heck of a difference in overcoming shelter-in-place*.*”*

Participants also noted the importance of close relationships as a source of emotional support and guidance.

“*I think pods are important … You know, have a group of people that you know and you trust.”*

“*It took me a while to get used to the idea of*… *asking for help*… *or opening up emotionally*… *So I think that helped create healthy relationships as well as more open ones to where that trust was created and it flourished*… *We [friends] definitely became closer*… *it was good to have that because I know even if it didn’t affect me like it was affecting them*, *it’s nice just to have someone to talk to about it*.*”*

#### 3. Community connection

Participants named many types of formal community structures that allowed them to connect with others and to both give and receive support. Examples included church, school, and substance use recovery groups. Participants noted the difference in being able to meet up with these supports in-person again after shelter-in-place policies ended.

“*I was starting to go to church again. I kind of missed it being absent for a while. I wouldn’t say I’m the most religious person ever–I’m really not… But I realized… it was always that community. We knew each other. We knew everyone at church… So I started doing that again. And honestly, I just got a lot happier when I saw people and started making an effort to be part of communities again.”*

“*I’m a recovering alcoholic and I’m in Alcoholics Anonymous*, *and I think one of my greatest recovery networks is my recovery network*… *Typically [they were] the same people*.*”*

Many social connections provided more than one function. In one example, a participant reported that gifts and other forms of instrumental support aided participants in feeling a sense of community and belonging.

“*And when we first moved here, someone anonymously gave me and my siblings each $1500 Visa gift cards to buy new clothes, so that was nice. I still don’t know who it was. I just know they’re from church… people were very welcoming, and they knew we were having a hard time, because it’s a rough transition, especially right in the beginning of the pandemic… people would bring us cookies or desserts. Just check in on us. And some of them would give us money to get a little treat or something.”*

## Discussion

The COVID-19 pandemic led to a sudden shift in social dynamics, and shelter-in-place policies changed social interactions dramatically. Here, we interviewed participants receiving mental health care to understand their perspectives on how social interactions and supports during shelter-in-place impacted their mental health recovery. We identified themes around the social experience and the types of social support that participants found helpful. Factors that influenced the social experience during shelter-in-place included participants’ baseline socialization patterns (such as pre-pandemic degree of introversion or extroversion, or pre-pandemic phase-of-life expectations), participants’ health concerns of the spread of COVID-19 through social interactions, and participants’ use of social media. Participants also identified three types of social supports that were helpful to their mental health, including social relationships that provided instrumental support, emotional support, and community connection. We review each theme below within an actionable framework for clinicians that evokes social prescribing practices.

### Baseline socialization patterns

Given the limitations of social activities during the pandemic, self-described introverts felt comfortable and enjoyed being able to stay at home in isolation, while some self-described extroverts reported feeling a loss of social connection that worsened their mental health. Individuals with more introverted tendencies might be more inclined to limit social activities and to avoid crowded gatherings. In addition, introverted individuals might be better equipped to cope with decreased social interaction while maintaining healthy behaviors and mindsets, without resorting to risky behaviors like substance use [18]. On the other hand, extroverts tend to have higher levels of social supports [19] and perceive their social relationships more positively [20], which could also help reduce the extent of loneliness during quarantine. Therefore, our findings suggest that clinicians consider personalizing social recommendations to individual preferences. For example, for an introvert, it can be helpful to emphasize time alone to recharge daily and to include self-care practices for mental wellbeing. For an extrovert, prioritizing interactions with positive individuals that foster healthy activities can be helpful advice for improving mental health.

### Health concerns about COVID-19 spread

Individuals with pre-existing health conditions or living with those with pre-existing conditions faced heightened risks during the pandemic, including increased susceptibility to severe illness from COVID-19. As a result, individuals living with someone with chronic disease seem to have adhered more strictly to shelter-in-place measures, even if at the detriment to their own social health. From a prescribing perspective, an informed discussion of health risks and benefits of social distancing should be considered in future pandemic scenarios, including reviewing risks to social wellbeing and how best to overcome these clinical gaps within appropriate bounds of public health guidelines.

### Use of electronic and social media communication

Social isolation during the pandemic led some participants to increase reliance on social media platforms to communicate with others. Our study showed a wide spectrum of social media use and its impact on mental health. Some participants reduced their use because it worsened their anxiety, and others found it helpful to continue connecting with others, find a community, and develop their hobbies. A national online survey with 3474 participants found that a decrease in mental health was associated with using social media for purposes to decrease loneliness and for entertainment motives, whereas mental health improved when social media was used for personal contact and maintaining relationships [21]. This suggests that the benefits of using social media may be partially influenced by the purposes of use and can be considered for patients to connect with their friends and family or to engage in their preferred hobbies and activities.

### Types of social supports for mental health

The *All of Us* study conducted by the National Institute of Health found that during the COVID-19 pandemic, several types of social supports were reported that closely mirrored the types identified by our study participants [6]. In particular, tangible support (similar to our instrumental support examples), emotional support, and positive social interactions (similar to our community connection theme) were identified as reducing the odds of depression symptoms in a dose-response manner. Indeed, we found that participants reported significant benefits to their wellbeing whenever they received assistance with tasks (like grocery shopping, medication delivery, or childcare,), or felt there was someone (a friend of family member) that they could speak to anytime, or felt connected to a wider community of people. Much like how clinicians perform reviews of symptoms or inventories of strengths, we propose that clinicians can catalog or checklist these domains of social support with patients to foster mental well-being and resiliency.

Our participants universally noted the importance of having a sense of community and a feeling of closeness with others as an important part of their wellness and resilience. Individuals can be encouraged to foster social connections, supportive relationships, and community engagement to mitigate the negative impact of social isolation on their mental health. We note that many participants found community organizations and structured identity group spaces important to their well-being during the pandemic. Church groups, recovery groups, and communities that form amongst colleagues are just a few examples of social spaces that helped people feel accepted and connected to others. Clinicians can consider these aspects of individuals’ identities and life experiences to encourage building relationships in ways that facilitate access to these social spaces. This concept is already being used in the realm of substance use recovery with the concept of “recovery capital,” or the internal and external sources that are necessary to achieve and sustain recovery [22].

### Limitations

This is the first qualitative study focused on patient perspectives of their social experience and the social supports that played a role in their mental health recovery during the early period of the COVID-19 pandemic. The framing of our study with a focus on social support may have attracted participants who felt positively toward their social life, and may not have captured perspectives from individuals who do not have as much social support. The majority of our participants were female, which may reflect the reality that women tend to have greater social connections than men [23], and thus may have been more likely to agree to participate in our study. Interviews were also done during 2023, a few years after the early pandemic era, and thus participants’ reflections may have been different had interviews taken place during the height of shelter-in-place. Nonetheless, we believe the timing of this study and the opportunity for reflection can also provide rich information regarding participants’ views of their social experiences. Lastly, we know that individuals of historically oppressed identities such as people of racial and ethnic minorities, sexual and gender-minority individuals, those with disabilities, rural residents, and immigrants and refugees, are at heightened risk for mental health challenges and loneliness [24]. It would be interesting for further studies to look more closely at the experiences of these populations in order to improve the quality of social prescribing and capture individuals’ specific needs.

## Conclusion

The COVID-19 pandemic has profoundly impacted the ways in which individuals engage in social relationships and their impact on mental health. Our qualitative research has highlighted several significant themes and factors influencing the social experience of the shelter-in-place period for individuals in mental health recovery and inform ways that we can structure social prescribing practices to better assist the needs of patients. Key factors to consider in social prescribing are individuals’ health concerns, baseline socialization patterns, use of social media, the types and frequency of communication, and encouraging a diverse set of social relationships that fulfill a variety of needs that are crucial to individuals’ mental health and well-being. The impact of these changes to social experience and social support due to shelter-in-place measures has varied, with individuals expressing both positive and negative sentiments toward the changes. This research contributes to a deeper understanding of the complexities of social dynamics during the COVID-19 pandemic. By recognizing the multifaceted nature of social experience and support, interventions and social prescribing practices can be tailored to individuals’ unique needs, promoting resilience, recovery, and well-being.

## Supporting information

S1 Fig Checklist. (DOCX)

S2 Fig Appendix. Study recruitment material.
(DOCX)

S3 Fig Appendix. Study interview guide.
(DOCX)
