# Supplementary material for: The impact of shelter-in-place during the COVID-19 pandemic on social support for mental health recovery: A prescribing-oriented qualitative study of patient perspectives
Source: PLoS One. 2025 Jan 17;20(1):e0316582. doi: 10.1371/journal.pone.0316582 (PMC11741564; doi:10.1371/journal.pone.0316582)
Supplement: S2 Fig — Study recruitment material. (DOCX) [file pone.0316582.s002.docx]

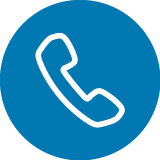

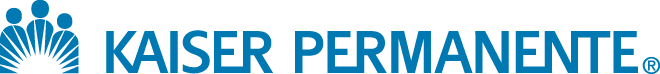


**What is the purpose?**

To investigate perspectives of the ways changes in social support affected mental health recovery during the COVID-19 pandemic.

**Am I eligible?**

If you are between ages 18 and 44, received mental health treatment at KP San Jose in the past year, active on KP.org, physically located in the Bay Area, and can read, speak, and write English then you may be eligible to participate. Eligibility will be determined during a brief telephone call.

**What is the duration?**

The study interviews will take about 70 minutes and we anticipate the entire study to be complete by 9/30/2024.

**Who do I contact?**

Dr. Hsin at **408-362-3750** if you have any questions, concerns or would like to volunteer to participate or opt out of the study.

# *Perspectives on Social Support during the COVID-19 Pandemic:*

# *Study Information Sheet*

If you are interested, please contact Dr. Honor Hsin at

408-362-3750.

## What will happen if I take part in this study?

You will participate in one 10-minute interview to determine eligibility, and if eligible, one 60-minute interview with the study team by Microsoft Teams meeting (which can be conducted by phone or computer). This interview will be recorded.

## Do I have to participate in the study?

Participation is completely voluntary. You are free to refuse, and your decision of whether to participate will not affect your medical care. If you decide to participate, you are free to change your mind and discontinue participation at any time. Already collected data cannot be withdrawn.

## What are the potential risks and discomforts?

Risks associated with this study are minimal. The only discomfort you may experience is feeling inconvenienced by having to complete the interview or answer personal questions; however, our research staff will make every reasonable effort to accommodate your schedule and will collect personal information respectfully and kindly.

## How will my privacy be protected?

Privacy is very important to us at Kaiser Permanente. Your name and any other information that allows you to be identified will not be stored in the same location as your research data and all data will be stored on the Kaiser Permanente Network. Investigators will ensure that the link between your name and these study numbers will never be released outside the hospital/study site unless required by law.

## Are there any benefits?

There are no direct benefits, but we hope that the results of this study may benefit you and other patients in the future.

## Will I be paid to take part in this study?

You will be compensated with a gift bag of Kaiser Permanente gift items (worth ~$30 in total value) after completion of your study interviews.

## Frequently

## Asked

## Questions:

SAN JOSE PSYCHIATRY
