# Supplementary material for: The impact of shelter-in-place during the COVID-19 pandemic on social support for mental health recovery: A prescribing-oriented qualitative study of patient perspectives
Source: PLoS One. 2025 Jan 17;20(1):e0316582. doi: 10.1371/journal.pone.0316582 (PMC11741564; doi:10.1371/journal.pone.0316582)
Supplement: S3 Fig — Study interview guide. (DOCX) [file pone.0316582.s003.docx]

**Interview Guide**

Thank you for participating in this study interview. Please take your time to respond to the questions. As a reminder, you may ask to pause the interview, skip questions, or stop the interview completely at any time. At the end of the interview, I’ll be asking you some demographic questions. Do you have any questions before we start?

**[Introduction]** The COVID-19 pandemic has fundamentally changed the way we interact with other people over the past several years, yet we know that human beings are resilient, and that often this resiliency comes from our connections to other people. We would like to know more about how the pandemic has changed the ways in which people connect with others, and the role this has on mental health and overall wellbeing.

For the purposes of this study, we will focus on two separate periods of time. The first we will call “shelter in place” - this is the period before the COVID-19 vaccines were developed, roughly the first year of the pandemic, during which the main way of combating the spread of COVID-19 was through local government policies encouraging people to stay at home and “socially isolate.” We want to hear about your experience during this time. The second period we will focus on is what we will call the “post-vaccine” period (April 2021 through the present).

**[Question 1]:** Tell me about a situation where someone or a group of people supported you greatly during a time of need. What was your relationship to this person or group, and how did they help you? Did you interact with them in-person or by phone or some other way or combination of ways? In the moments when they were supporting you, how did that make you feel?

**[Question 2**] Let’s think about your mental health before the COVID-19 pandemic began. I’m going to read a list of categories of people who may have been supportive in your life. For each category, can you think of people in your life who were supportive? How were they helpful? How did they make you feel? How did you interact with them [probe: frequency, duration, place, modality]?

- Significant others?
- Family?
- Friends?
- School classmates?
- Work colleagues?
- Community or church groups?
- Online groups?
- Dating apps?
- Other?
- [If no response: It’s OK if you can’t think of something in this category.]

**[Question 3]** The COVID-19 pandemic changed a lot of ways we interact with people. I want to better understand how COVID changed the way you socialized. What were some changes to the way you interacted with others that you noticed? [probe: did this happen during social isolation? Did this change in the post-vaccine period?]

**[Question 4]** Were there social supports that you gained during the COVID-19 pandemic? Were there social supports that you lost? How did these losses and gains affect your mental health?

**[Question 5]** Knowing your experience of shelter-in-place, how do you think you might “prepare socially” if another shelter-in-place pandemic occurs? What would you advise to others to “prepare socially” for a future pandemic?

**[Question 6]** Do you feel that you “missed out” on a social experience or social interaction you otherwise would have had? How do you feel about this? Is there anything that you feel helped make up for it?

**[Question 7]** People stay connected socially in lots of different ways – meeting up in person, phone calls, social media, emails or texting, etc. Can you name all the different ways you connect with others? *[If they don’t mention one of the formats below, can ask them if they use that format; for example, “Do you use email as a way of staying connected?”]*

- Texting
- Meeting in-person
- Talking on the phone
- Virtual face-to-face (Facetime, Zoom, etc)
- Virtual group face-to-face (Facetime, Zoom, etc)
- Emails
- Personal social media platforms (Facebook, Twitter, Instagram, Tiktok, etc)
- Anonymous social media (Reddit, message boards, etc)

How did these ways change during shelter-in-place, and how did they change after the guidelines loosened up? How did you feel about these changes? How do you think these changes affected your mental health and wellbeing?

--------------------------------------------

**[Demographics]**

Thank you for sharing your experiences and answering these questions. Last, we would like to learn some basic information about you. This part should only take a few minutes of our remaining interview time. You may skip any questions you wish.

What is your age?

What is your identified gender?

What is your identified race and/or ethnicity?

What is your highest level of education?

Which of the following psychiatry department services have you ever used at Kaiser Permanente San Jose (please choose as many as applicable):

- Outpatient medication management or appointments with a psychiatrist for medications?
- Outpatient individual psychotherapy or appointments with a therapist for counseling?
- Outpatient group therapy or outpatient treatment program?
- Intensive outpatient program, meaning more than one treatment contact per week?
